# Impact of the Covid-19 Pandemic on Youth Mental Health

**DOI:** 10.1192/j.eurpsy.2022.1228

**Published:** 2022-09-01

**Authors:** A. Hvolby, J.P. Hansen

**Affiliations:** 1 Psychiatri in region of Southern Denmark, Child And Adolescent Psychiatric Department South Jutland, Esbjerg N, Denmark; 2 psychiatry in region of Suothern Denmark, Psychiatric Department, Esbjerg, Denmark

**Keywords:** Child and adolescent psychiatry, Covid-19, Virtual consultation

## Abstract

**Introduction:**

The COVID-19 pandemic resulted in national lockdowns in several countries. Previous global epidemics led to an increase in the number of psychiatric patients presenting symptoms of anxiety or depression. Knowledge about the impact of early lockdown initiatives during the COVID-19 pandemic on the number of healthcare interactions is sparse.

**Objectives:**

To investigate both the impact of the Danish lockdown event on psychiatric patients’ contact with the healthcare system, stratified by type of contact (face-to-face (FTF) or virtual) and ICD-10 diagnosis, and how acute contacts were impacted in the five regions in Denmark.

**Methods:**

Contacts in this study include all recorded FTF and virtual treatment interactions between patients and healthcare systems. An interrupted time series analysis was applied to determine the effect of the COVID-19 lockdown event on the number of contacts with psychiatric hospitals in Denmark, from February 25, 2019 to May 3, 2020. The analyses took a Box-Jenkins approach to fit an autoregressive integrated moving average (ARIMA) model.

**Results:**

Virtual contacts replaced most FTF contacts during the lockdown. For most patient groups, the total number of contacts did not decrease significantly. However, for child and adolescent patients diagnosed with F 10–19, 70–79, and 80–89, the number of contacts decreased during lockdown. The number of acute contacts with the psychiatric system decreased significantly during lockdown.

**Conclusions:**

The Danish healthcare system was forced to introduce innovative tele-psychiatry to mental health care during the lockdown. Disruption to service delivery was minimized because the resources were in place to sustain the transition from FTF to virtual contacts.

**Disclosure:**

No significant relationships.

